# Relationship between clinical-epidemiological parameters and outcomes of patients with COVID-19 admitted to the intensive care unit: a report from a Brazilian hospital

**DOI:** 10.3389/fpubh.2023.1241444

**Published:** 2023-09-22

**Authors:** Maisah Meyhr D’Carmo Sodré, Uener Ribeiro dos Santos, Heitor Portella Povoas, Júlio Lenin Guzmán, Caroline Junqueira, Tayana Oliveira Trindade, Sandra Rocha Gadelha, Carla Cristina Romano, Aline Oliveira da Conceição, Eduardo Gross, Aline Silva, Rachel Passos Rezende, Renato Fontana, Camila Pacheco Silveira Martins da Mata, Lauro Juliano Marin, Luciana Debortoli de Carvalho

**Affiliations:** ^1^Department of Biological Sciences, Santa Cruz State University, Ilhéus, Bahia, Brazil; ^2^Hospital de Ilhéus, Ilhéus, Bahia, Brazil; ^3^Program in Cellular and Molecular Medicine, Boston Children's Hospital, Boston, MA, United States; ^4^Department of Pediatrics, Harvard Medical School, Boston, MA, United States; ^5^René Rachou Institute, Oswaldo Cruz Foundation, Belo Horizonte, Minas Gerais, Brazil; ^6^Laboratory of Microbiology, Risoleta Tolentino Neves Hospital, Belo Horizonte, Minas Gerais, Brazil; ^7^Department of Health, Santa Cruz State University, Ilhéus, Bahia, Brazil

**Keywords:** COVID-19, SARS-CoV-2, biomarkers, epidemiology, SAPS3, in-hospital mortality, intensive care unit, catheter

## Abstract

**Background:**

People in low-income countries, especially those with low socio-economic conditions, are likelier to test positive for SARS-CoV-2. The unequal conditions of public health systems also increase the infection rate and make early identification and treatment of at-risk patients difficult. Here, we aimed to characterize the epidemiological profile of COVID-19 patients in intensive care and identify laboratory and clinical markers associated with death.

**Materials and methods:**

We conducted an observational, descriptive, and cross-sectional study in a reference hospital for COVID-19 treatment in the Southern Region of Bahia State, in Brazil, to evaluate the epidemiological, clinical, and laboratory characteristics of COVID-19 patients admitted to the intensive care unit (ICU). Additionally, we used the area under the curve (AUC) to classify survivors and non-survivors and a multivariate logistic regression analysis to assess factors associated with death. Data was collected from the hospital databases between April 2020 and July 2021.

**Results:**

The use of bladder catheters (OR 79.30; *p* < 0.0001) and central venous catheters (OR, 45.12; *p* < 0.0001) were the main factors associated with death in ICU COVID-19 patients. Additionally, the number of non-survivors increased with age (*p* < 0.0001) and prolonged ICU stay (*p* < 0.0001). Besides, SAPS3 presents a higher sensibility (77.9%) and specificity (63.1%) to discriminate between survivors and non-survivor with an AUC of 0.79 (*p* < 0.0001).

**Conclusion:**

We suggest that multi-laboratory parameters can predict patient prognosis and guide healthcare teams toward more assertive clinical management, better resource allocation, and improved survival of COVID-19 patients admitted to the ICU.

## 1. Introduction

The global impact of the coronavirus disease 2019 (COVID-19) is unquestionable. Concerning deaths, 68% were concentrated in 10 countries: Brazil, Egypt, India, Indonesia, Mexico, Peru, Russia, South Africa, Turkey, and the United States (1). The first COVID-19 case in Brazil was confirmed on February 26, 2020. The disease rapidly spread in the capital and countryside regions, and within a month, community transmission was documented in Brazilian cities. Bahia is Brazil’s fourth most populous State and the sixth state in cumulative deaths as of 2022, with the first case confirmed on March 6, 2020, through reverse transcription-quantitative polymerase chain reaction (RT-qPCR) (2–4).

By December 2022, Brazil had an incidence coefficient of 17,152, with an incidence rate of 11,738 per 100,000 inhabitants in Bahia. One of the main cities in the Southern Region of Bahia State, Ilhéus, has an incidence rate of 17,129 per 100,000 inhabitants, which is higher than that of Bahia. While Brazil’s lethality rate is 1.9%, Bahia’s rate is 1.8%, and Salvador, Vitória da Conquista, Feira de Santana, and Ilhéus have the highest number of deaths (2, 5).

Generally, the infection can manifest in a varied clinical spectrum ranging from asymptomatic to critical presentations. In addition to respiratory symptoms, severe cases may present with extrapulmonary complications or multiple organ failure, and early identification and treatment of at-risk patients are essential to prevent mortality (6–8). From an epidemiological perspective, a profile analysis of severe COVID-19 cases indicates that males have higher mortality rates than females do. Furthermore, comorbidities such as hypertension, diabetes, heart disease, malignancy, and immunodeficiency are more prevalent in individuals with severe COVID-19, irrespective of sex (9–13). However, in the Southern region of Bahia State, at the beginning of the pandemic, males with comorbidities were more likely to test positive for SARS-CoV-2 (14).

Early indicators of death in hospitalized patients guide clinical decision-making and include blood pressure, respiratory rate, D-dimer levels, international normalized ratio (INR), **and Simplified** Acute Physiology Score 3 (SAPS 3), which are predictors of in-hospital mortality (15–20). Multiple biomarkers are necessary to assess disease progression and an individual’s response to clinical interventions (21–23). Notably, well-established biomarkers include interleukin-6 (IL-6) and C-reactive protein levels (24–26).

Nonetheless, the profile of SARS-CoV-2 infection changes as new variants emerge, increasing the infection rate, mortality, and symptomatic profile (27–29). People in low-income countries, especially those with low socio-economic conditions, are more likely to test positive for SARS-CoV-2, with higher mortality rates (30). Accordingly, a study conducted in South America showed high seropositivity in individuals with low socio-economic status (31). Similarly, the unequal conditions of public health systems increase the infection rate and make early identification and treatment of at-risk patients difficult (32–34).

We aimed to characterize the clinicopathological profile of hospitalized patients with COVID-19 admitted to the intensive care unit (ICU) of a reference hospital for COVID-19 in the Southern Region of Bahia State, in Brazil, between April 2020 and July 2021. Additionally, we analyzed the data to identify the laboratory and clinical markers associated with death in patients admitted to the ICU.

## 2. Materials and methods

### 2.1. Ethical considerations

The study was submitted to the Research Ethics Committee of the State University of Santa Cruz and approved under protocol number CAAE:40671720.4.0000.5526 on February 22, 2021.

### 2.2. Study design, data collection, and curation

We conducted an observational, descriptive, and cross-sectional study at a reference hospital for COVID-19 treatment in the Southern Region of Bahia State, Brazil. Data from individuals admitted to the ICU with COVID-19, confirmed using RT-qPCR for SARS-CoV-2 RNA, were collected between April 2020 and July 2021. The care and clinical observations of the patients were performed by a multidisciplinary team at the hospital, and a registered nurse entered the epidemiological, clinical, and complete laboratory information into the Epimed Monitor System database as a hospital routine. The Epimed Monitor System is a cloud-based registry of clinical and administrative data for managing intensive care unit patients.

The patient data were collected from the Epimed Monitor System database. No patient identification was accessed; instead, patients were identified through numerical coding, ensuring the confidentiality and anonymity of participants. The inclusion criteria for this study were as follows: individuals who entered the ICU between April 2020 and July 2021, adults (18 years or older), positivity for SARS-CoV-2 RNA by RT-qPCR, at least 1 day (24 h) of ICU stay, and availability of clinical and epidemiological data in the Epimed Monitor System database. The exclusion criteria included: patients aged <18 years, those who tested negative or inconclusive for SARS-CoV-2 RNA by RT-qPCR, and those who stayed in the ICU for less than 24 h. The clinical data considered for the analysis included arterial hypertension, diabetes, vasopressor use, renal injury, and respiratory failure. Laboratory data included the fraction of inspired oxygen (FiO_2_), partial pressure of carbon dioxide (PaCO_2_)/FiO_2_, serum lactate, arterial pH, serum creatinine (CR), serum urea (SR), and white blood cell count (measured as white blood cell,WBC, count × 1,000/mm^3^). Additionally, invasive procedures associated with severe cases, such as mechanical ventilation and catheter use, were included in the analysis. We considered all COVID-19-positive individuals admitted to the ICU whose epidemiological, clinical, and laboratory data were available during the study period.

**Figure 1:**
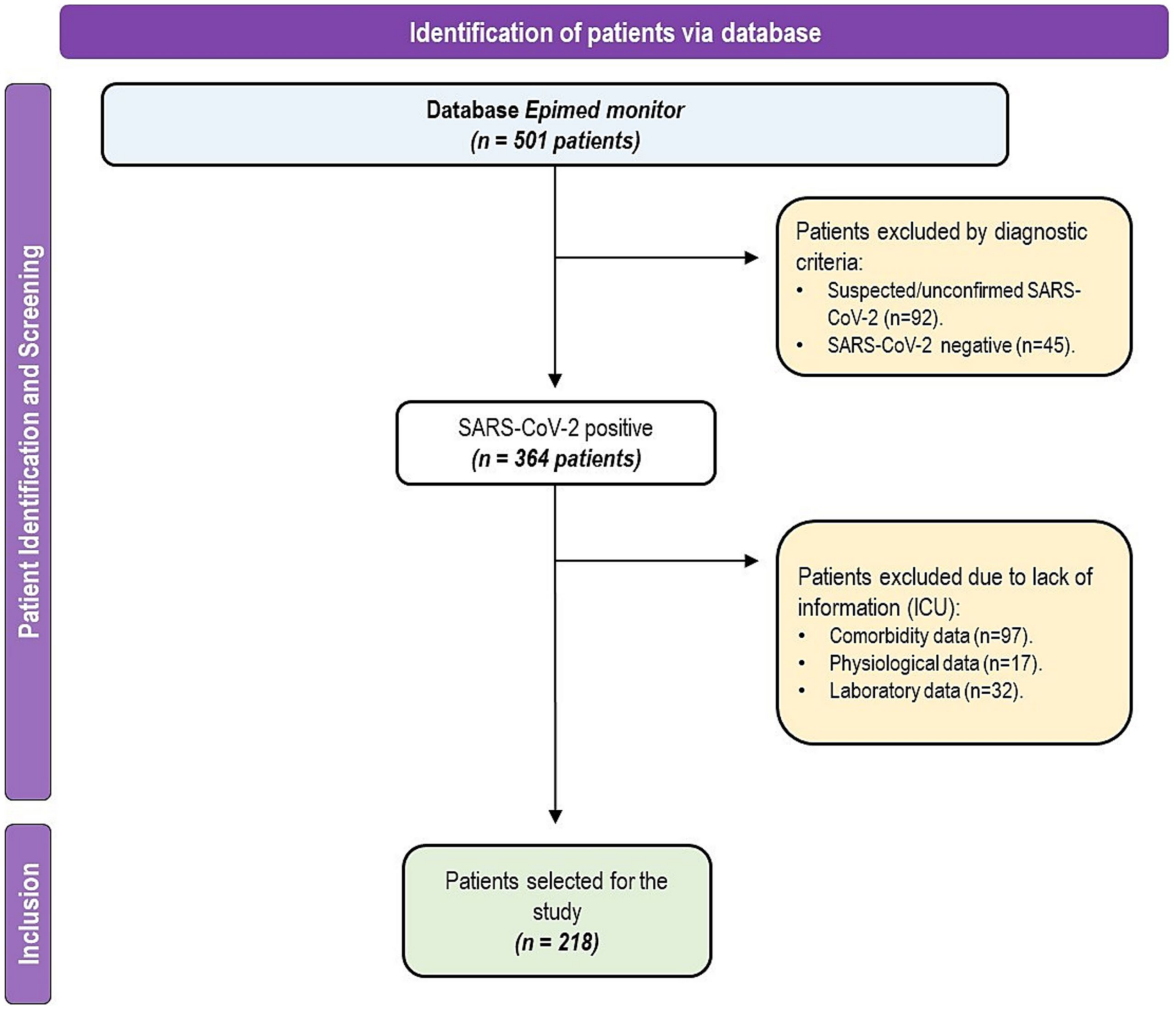
Participant selection flowchart in the reference hospital database.

In total, 501 individuals were included in the analysis. We excluded individuals who tested negative for SARS-CoV-2 (*n* = 92) and those with suspected or unconfirmed detection (*n* = 45) by RT-qPCR. Individuals with incomplete data on comorbidities (*n* = 97), physiological data (*n* = 17), or laboratory data (*n* = 32) were excluded from the analysis (Figure 1). In total, 218 individuals were included in this study.

### 2.3. Breakdown of variables for the study

Categorical variables: Hypertension, diabetes, vasopressors, renal injury, respiratory failure, invasive and non-invasive mechanical ventilation, and use of catheters are represented as absolute frequencies (n), percentages (%), odds ratios (ORs), and 95% confidence intervals (95% CIs) with respective *p*-values. Continuous variables included: blood urea nitrogen (BUN), CR, age, lactate, white blood cells (WBCs), greater PaCO_2_, greater PaFiO_2_, greater PaO_2_/PaFiO_2_, greater arterial pH, hospital stay, ICU stay, SAPS3, and urea, are shown as individual values, mea*n* ± standard deviation, minimum, maximum, and median values, with respective *p*-values. For the logistic regression model, data were represented as ORs and 95% confidence intervals (CI associated with individual *p*-values).

### 2.4. Statistical analyses

Continuous variables were evaluated for normality using the Shapiro–Wilk test. Variables that assumed a normal distribution and those that did not were analyzed using Student’s *t*-test and the Mann–Whitney *U* test, respectively.

We classified survivors and non-survivors using the area under the curve (AUC) from the Wilson/Brown method, with sensitivity (Se, %), specificity (Sp, %), and 95% CI values associated with the respective *p*-values. First, the cut-off point of the variables (discriminant value) was established as the value associated with maximum sensitivity and specificity (28). The statistical significance of the cut-off point was then selected by analyzing the sensitivity and specificity, AUC, value of *p*, and 95% CI values (35).

We used Pearson’s chi-square test (*X*^2^) and Fisher’s exact test (36) to analyze the association between the frequency of each categorical variable and the participants’ clinical outcomes (ICU discharge and death). Statistical analyses were performed using the GraphPad Prism software (version 9.0; GraphPad Prism Software, San Diego, CA, United States) at a significance level of 5%. Therefore, *p < 0.05* were considered statistically significant.

We used a bivariate analysis with a significance level of *p* < *0.20* to identify candidate variables to fit in the logistic regression analysis in a multivariate model. Moreover, a *stepwise backward* (conditional) elimination method was used, and the best model was defined as one that included statistically significant variables (*p < 0.05*) and minimized the value of the Akaike Information Criteria (AIC). All the statistical analyses were performed using Statistical Package for Social Sciences (SPSS) version 26.0 (IBM Corporation, Armonk, NY, United States). Los Angeles, CA, United States.

## 3. Results

### 3.1. Clinical profile of COVID-19 patients associated with patient outcome

**Figure 2:**
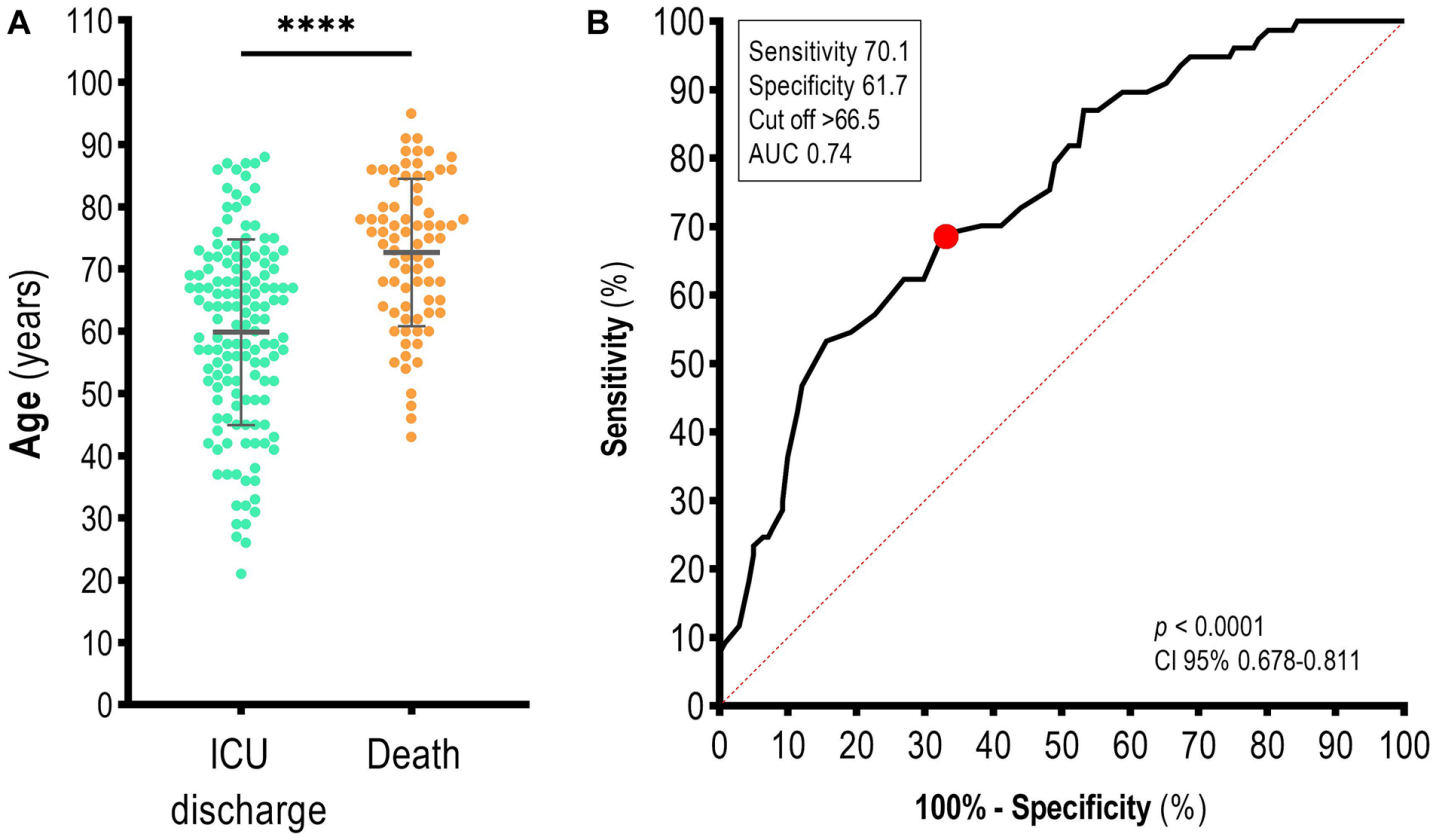
Age distribution of patients with COVID-19 in the ICU of a reference hospital in the Southern Region of Bahia State, Brazil. **(A)** Age in years and **(B)** the area under the curve (AUC) to discriminate between survivors and non-survivors. The red point indicates the cut-off value. Mann–Whitney test. Data are presented as the mea*n* ± standard deviation. *p*-values <0.05 were considered statistically significant. ****p < 0.0001.

Between April 2020 and July 2021, 501 SARS-CoV-2 positive individuals from the Southern Region of Bahia State were admitted to the ICU of a referred hospital for COVID-19 treatment. After screening the data, 218 individuals were included in our analysis:141 (64, 68%) were discharged from the ICU, and 77 (35, 32%) died. The average age of patients was 64.37 ± 15.19 years, and males comprised the majority of our population (*n* = 123, 56.4%). Sex did not increase the odds of death (OR 1.58; 95% CI 0.89–2.81; *p* = 0.112), while patients with advanced age were more likely to die (Figure 2A); accordingly, the concentration of non-survivors was higher among patients older than 66 years of age (Se 70.1; Sp 61.7; AUC 0.74; 95% CI 0.678–0.811; *p* < 0.0001; Figure 2B). Furthermore, among clinical requirements in hospitalized patients, the use of vasopressors (OR 6.28; 95% CI 3.08–12.56; *p* < 0.0001) and mechanical ventilation (OR 5.56; 95% CI 3.05–10.15; *p* < 0.0001) increased the odds of death (Table 1). We also observed that the use of a bladder catheter (*p* < 0.0001), central venous catheter (*p* < 0.0001), and arterial by 79.30, 45.12, and 16.11, respectively. On the other hand, the use of non-invasive mechanical ventilation decreased the chance of death in hospitalized patients (OR 0.34; 95% CI 0.18–0.60; *p* = 0.0003).

**Table 1:** Clinical characteristics of COVID-19 patients admitted to the ICU.

	COVID-19 patients			Univariate analysis		
	Total *n* = 218 (%)	Death *n* = 77 (%)	ICU discharge *n* = 141 (%)	OR	95% CI	*p^a^*
Sex						
Male	123 (56.4)	49 (22.5)	74 (33.9)	1.58	0.89–2.81	*0.112*
Female	95 (43.6)	28 (12.9)	67 (30.7)			
Arterial hypertension						
Yes	173 (79.3)	63 (28.9)	110 (50.5)	1.27	0.61–2.61	*0.507*
No	45 (20.7)	14 (6.4)	31 (14.2)			
Diabetes						
Yes	92 (42.2)	34 (15.6)	58 (26.6)	1.13	0.66–2.00	*0.669*
No	126 (57.8)	43 (19.7)	83 (38.1)			
Vasopressors						
Yes	43 (80.3)	30 (13.7)	13 (6.0)	6.28	3.08–12.56	***<0.0001***
No	175 (19.7)	47 (21.6)	128 (58.7)			
Kidney injury						
Yes	7 (3.2)	5 (2.3)	2 (0.9)	0.21	0.04–1.01	*0.099*
No	211 (96.8)	72 (33.2)	138 (63.6)			
Respiratory failure						
Yes	208 (96.3)	76 (34.9)	132 (60.5)	5.18	0.82–57.58	*0.102*
No	10 (3.7)	1 (0.5)	9 (4.1)			
Mechanical ventilation						
Yes	78 (35.8)	47 (21.5)	31 (14.2)	5.56	3.05–10.15	***<0.0001***
No	140 (64.2)	30 (13.8)	110 (50.5)			
Non-invasive mechanical ventilation						
Yes	92 (42.2)	20 (9.2)	72 (33.0)	0.34	0.18–0.60	***<0.0003***
No	126 (57.8)	57 (26.1)	69 (31.7)			
Central venous catheter						
Yes	139 (63.8)	75 (34.4)	64 (29.4)	45.12	11.60–191.2	***<0.0001***
No	79 (36.2)	2 (0.9)	77 (35.3)			
Arterial catheter						
Yes	124 (56.9)	70 (31.1)	54 (24.8)	16.11	7.05–39.07	***<0.0001***
No	94 (43.1)	7 (3.2)	87 (39.9)			
Bladder catheter						
Yes	145 (66.5)	76 (34.9)	69 (31.6)	79.30	13.693–810.2	***<0.0001***
No	73 (33.5)	1 (0.5)	72 (33.0)			

^a^Chi-Square test (*X*^2^) and Fisher’s exact test. Highlighted values are considered statistically significant.

### 3.2. Time of ICU stay and clinical score can be used to discriminate survivors and non-survivors with COVID-19 in the ICU

**Figure 3:**
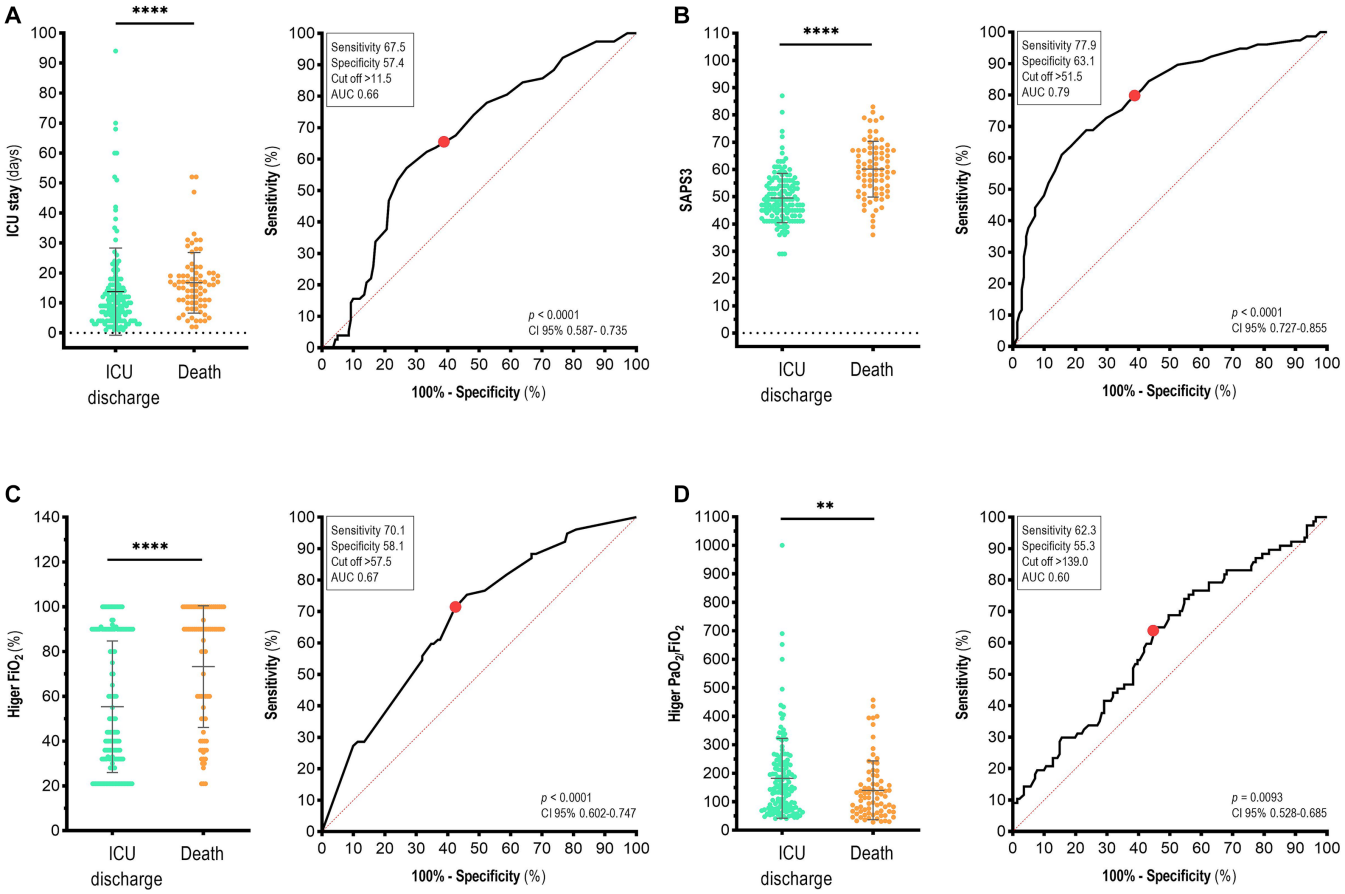
Clinical parameters used to discriminate between survivors and non-survivors with COVID-19 in the ICU of a reference hospital in the Southern Region of Bahia State, Brazil. Analysis of variables (left) and area under the curve (AUC, right) for **(A)** ICU stay, **(B)** SAPS3 – Simplified Acute Physiology Score 3, **(C)** higher FiO2, and **(D)** higher PaO2/FiO2. The red point indicates the cut-off value. Mann–Whitney test. Data are presented as the mea*n* ± standard deviation. *p*-values <0.05 were considered statistically significant. **p < 0.01; ****p < 0.0001.

Hospitalized patients presented an average ICU stay of 14.8 ± 13.18 days. Differences between the length of ICU stay of the patient discharged (13.77 ± 14.51) and death (16.71 ± 10.11) were observed (*p* < 0.0001; Figure 3A, left). Furthermore, individuals with an ICU stay of >11.5 days were more likely to die (Se 67.5; Sp 57.4; AUC 0.66, 95% CI 0.587–0.735; *p* < 0.0001; Figure 3A, right).

The SAPS3 is a scoring system widely used to predict in-hospital mortality and uses pertinent variables of acute physiological derangements, current conditions, interventions, and health status before ICU admission to predict mortality (24, 25). The highest concentration of deaths due to COVID-19 was in ICU participants who had SAPS3 > 51.5 (Se 77.9; Sp 63.1; AUC 0.79; 95% CI 0.727–0.855; *p < 0.0001;* Figure 3B). Moreover, PaO_2_/FiO_2_ was used to determine the need for invasive or non-invasive mechanical ventilation in the hospital setting and was associated with death. Participants with FiO_2_ greater than 57.5% were more likely to die (Se, 70.1; Sp, 58.1; AUC, 0.67; 95% CI 0.602–0.747; *p < 0.0001*; Figure 3C). Regarding the PaO_2_/FiO_2_ ratio, which represents the degree of lung injury, participants with a higher PaO_2_/FiO_2_ ratio > 139.0 were more likely to die (Se 62.3; Sp 55.3; AUC 0.60; 95% CI 0.528–0.685; *p = 0.0093;* Figure 3D). Among other markers such as Higher PaCO_2_, PaO_2_, lower diastolic blood pressure, lower systolic blood pressure, and hospital stay (days), we did not observe any statistical significance (Supplementary Figures S1A–E).

### 3.3. Laboratorial markers associated with death in COVID-19 patients in the ICU

**Figure 4:**
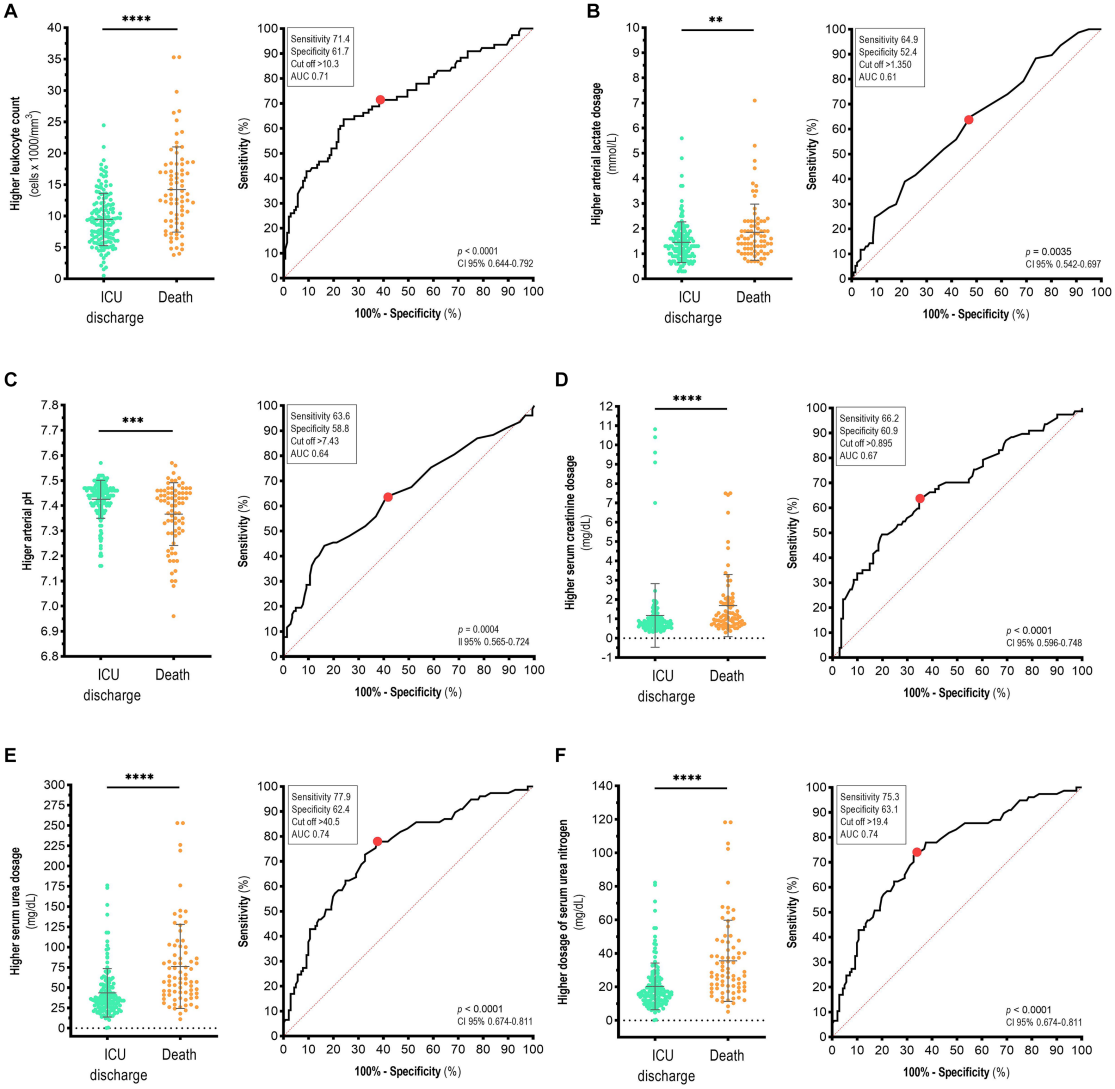
Biochemical and hematological parameters used to discriminate between survivors and non-survivors with COVID-19 in the ICU of a reference hospital in the Southern Region of Bahia State, Brazil. Analysis of variables (left) and area under the curve (AUC, right) for **(A)** higher leukocyte count, **(B)** higher arterial lactate, **(C)** higher arterial pH, **(D)** higher serum creatinine, **(E)** higher serum urea, and **(F)** serum urea nitrogen. The red point indicates the cut-off value. Mann–Whitney test. Data are presented as the mea*n* ± standard deviation. *p*-values <0.05 were considered statistically significant. **p < 0.01; ***p < 0.001; ****p < 0.0001.

COVID-19 patients who died in the ICU presented higher leukocyte count (14.22 ± 6.78 cells x 1,000/mm^3^) than did the ICU-discharged patients (9.46 ± 4.18 cells x 1,000/mm^3^; *p* < 0.0001; Figure 4A, left). Although cardiovascular complications and thromboembolism have been previously reported in COVID-19 patients (37, 38), we did not observe a difference in the platelet count between dead and discharged patients with COVID-19 in the ICU (Supplementary Figure S1F). Among the studied biomarkers, we observed higher arterial lactate (*p < 0.01*), serum creatine (*p < 0.0001*), serum urea (*p < 0.0001*), and serum urea nitrogen (*p < 0.0001*) in patients with death outcomes than in discharged patients (Figures 4B,D–F, left), whereas higher arterial pH was lower in death patients (*p* < 0.001; Figure 4C, left). Higher leukocyte count (Se 71.4; Sp 61.7; AUC 0.71; 95% CI 0.644–0.792; *p* < 0.0001; Figure 4A, right), serum creatine (Se 77.9; Sp 62.4; AUC 0.74; 95% CI 0.674–0.811; *p* < 0.0001; Figure 4E, right), and serum urea nitrogen (Se 75.3; Sp 63.1; AUC 0.74; 95% CI 0.674–0.811; *p* < 0.0001; Figure 4F, right), were the best markers to discriminate survivors and non-survivors.

### 3.4. Factors associated with death from COVID-19 patients in the ICU

We performed multivariate logistic regression analysis to verify whether the significant variables described above were associated with death in COVID-19 patients. The analysis revealed that men were more likely to die from COVID-19 in the ICU (OR 2.73; 95% CI 1.15–6.46; *p* = 0.022; Table 2). Moreover, the ICU stay and PCO_2_ did not increase the odds of death in our population, while bladder catheter (OR 28.09; 95% CI 2.69–292.8; *p* = 0.005) and central venous catheter (OR 12.97; 95% CI 2.25–74.74; *p* = 0.004) presented as risk factors and increased the odds to death (Table 2).

**Table 2:** Logistic regression analysis of characteristics associated with death in COVID-19 patients.

	Univariate analysis			Multivariate analysis^‡^		
	OR	95% CI	*p*	OR	95% CI	*p*
Age, years	1.07	1.04–1.10	***0.000***	1.08	1.04–1.11	***0.000***
Sex						
Female	Reference			Reference		
Male	1.58	0.89–2.80	*0.113*	2.73	1.15–6.46	***0.022***
Bladder catheter						
No	Reference			Reference		
Yes	79.30	10.73–586.13	***0.000***	28.09	2.69–292.8	***0.005***
ICU stay, days	1.01	0.99–1.03	*0.122*	0.97	0.94–1.00	*0.106*
Central venous catheter						
No	Reference			Reference		
Yes	45.11	10.65–190.97	***0.000***	12.97	2.25–74.74	***0.004***
PCO_2_, mmHg	1.01	0.99–1.03	*0.097*	0.97	0.95–1.00	*0.082*
White blood cell count, 10^3^/mm^3^	1.18	1.11–1.26	***0.000***	1.17	1.07–1.27	***0.000***

^‡^Multivariate logistic regression model with stepwise backward (conditional) elimination method.

OR, odds ratio; 95% CI, confidence interval 95%. Highlighted values are considered statistically significant.

## 4. Discussion

Herein, we describe the epidemiological and clinical characteristics of COVID-19 patients admitted to the ICU of a hospital for COVID-19 treatment in the Southern Region of the Bahia State, Brazil. We also analyzed the factors associated with death. For example, we identified clinical parameters such as the use of mechanical ventilation, central venous catheters, arterial catheters, vasopressors, and bladder catheters related to the respiratory, cardiovascular, and urinary systems, which increased the odds of death in COVID-19 patients in intensive care.

The impact of the COVID-19 pandemic has not been homogeneous worldwide, with some countries being more affected and presenting different mortality rates (1). Social factors and precarious socio-economic conditions are drivers of increased infection and mortality rates (30–34, 39, 40). For example, the positivity of SARS-CoV-2 infection in cities in the Southern Region of Bahia State was negatively correlated with a low Human Development Index (HDI) (41) (Bahia State has a low HDI, and the average worker salary is less than US$ 600.00). Furthermore, it was also shown that individual and community risk factors for SARS-CoV-2 infection varied between the Bahia cities; for example, gender and age were not homogenous risk factors for SARS-CoV-2 infection between the 12 cities studied (42).

A retrospective study in Brazil using population-based registers demonstrated that individuals hospitalized for less than 4 days presented high odds of death (OR 2.07, 95% CI 2.05–2.10). Moreover, the odds of death were five times higher than for individuals requiring ICU admission (OR 5.19, 95% CI 5.14–5.24) (43). Notably, in our study, 35.32% of the COVID-19 patients in the ICU died. An in-hospital mortality rate of 37% for COVID-19 was reported in Brazil, and the mortality rate increased with advanced age, low education level, comorbidities, and in individuals of black/brown self-reported race (44).

Notably, determining the clinical-epidemiological and laboratory profiles of COVID-19 patients can provide valuable information for a multidisciplinary healthcare team for more assertive clinical management, better resource allocation, and improved survival of patients admitted with COVID-19 in the ICU (44, 45). In this study, when multifactorial variables were correlated using regression analysis, the male sex had a higher chance of death, consistent with previous studies (46, 47). Furthermore, male-specific variables such as hypogonadism and low testosterone levels have been linked to the development of comorbidities that increase mortality from COVID-19, including type 2 diabetes, obesity, and cardiovascular disease (46). Additionally, evidence suggests that unbalanced testosterone levels may facilitate infection and disease progression in men because of their impact on the expression of the SARS-CoV-2 receptor, angiotensin-converting enzyme-2, and major fusogenic transmembrane serine protease 2 under regular transcription by androgens (47, 48).

The average age of the ICU patients in our study was 64 years. We observed that older patients, especially those aged >66 years, were more likely to die from COVID-19. Comparing patients from wards and ICU, Pereira and coauthors showed that mortality rates increased with advanced age, according to sex, ethnic/racial background, and vaccination status (43). Moreover, previous studies have indicated that individuals aged 65 years and older have a higher risk of death from COVID-19 (49, 50). This may be attributed to an age-related decline in innate immunity and immunosenescence. Accordingly, a study conducted in 2020 in the Southern Region of Bahia State with hospitalized patients showed a higher frequency of COVID-19 among patients of advanced age (51). Furthermore, in severe cases of COVID-19, hematological changes in peripheral leukocytes reflect a compromised immune response during SARS-CoV-2 infection. These changes are early indicators of fatal outcomes and are crucial for maintaining immune homeostasis during viral infections (52).

In addition, the current study also suggests a high WBC count >10.03 cells x 1,000/mm^3^ as a predictive death parameter, which was higher in patients with death outcomes than in discharged patients. These data are consistent with those of previous studies (53) and a meta-analysis examining the relationship among WBC count, COVID-19 severity, and mortality (54). The meta-analysis reported a WBC count of 0.41 × 10^9^ /L for patients with moderate COVID-19, while the count increased significantly to 4.15 × 10^9^ /L in patients who died (55). Another meta-analysis showed that the WBC and neutrophil counts decreased significantly in patients with mild COVID-19. However, similar to the results of the present study, higher counts were observed in severe COVID-19 (56).

Although we have shown that clinical parameters such as the use of mechanical ventilation, central venous catheters, arterial catheters, vasopressors, and bladder catheters increased the odds of death in COVID-19 patients, we also analyzed laboratory markers, including arterial lactate, serum creatine, urea, and serum urea nitrogen, which were higher in patients who died than in those discharged from the ICU. Investigators have suggested that determining changes in lactate levels can provide insights into COVID-19 pathophysiology and multisystem interactions (57). Furthermore, oxygen deprivation in tissues leads to lactate overproduction because pyruvate cannot be oxidized in the Krebs cycle. Predisposing factors for lactic acidosis, including diabetes and acute respiratory distress syndrome are common in hospitalized COVID-19 patients. In addition, COVID-19-related damage to alveolar cells may contribute to increased lactic acid (22, 58, 59).

Due to altered dyspnea and extremely low oxygen saturation, individuals with impaired respiratory metabolism are at an exceptionally high risk of death. Specifically, changes in carbon dioxide levels trigger a hypoxic threshold, resulting in lung damage. Under normal hypoxic conditions, even a slight imbalance in PaCO_2_ levels quickly evokes significant increases in ventilation per minute and brief respiratory alkalosis, which physiologically alters blood pH (37, 46, 47).

Regarding laboratory markers, we also observed an association between COVID-19 non-survivors and urea and serum creatinine levels. These biomarkers can help evaluate kidney injury, especially the acute forms that occur in 3–29% of COVID-19 patients. According to a study of 701 patients with COVID-19, both kidney injury and acute kidney injury increased the risk of death, with elevated serum creatine and urea nitrogen levels being predictive of mortality (60). Furthermore, we observed high levels of these biomarkers in COVID-19 patients who died. Data from 95 patients, of whom 25 were admitted to the ICU, showed a short-term increase in the urea and serum creatine ratios (OR, 1.72; 95% CI, 1.20–2.66), characterizing them as independent predictors of the prognosis of death.

Finally, we observed that the odds of death were five times higher for individuals requiring mechanical ventilation in the ICU, and patients with a higher FiO_2_ were more likely to die of COVID-19 in the ICU. During the COVID-19 outbreak, ICU stay and mechanical ventilation devices have been associated with respiratory failure (61–66), and the unprecedented number of patients weaned from non-invasive ventilation proved to be highly challenging. Additionally, here, the bladder catheter and central venous catheter groups presented higher ORs for death, 79.3 and 45.12, respectively.

Invasive ventilation is an intricate procedure that requires skilled multidisciplinary teams and expensive equipment. The lack of trained professionals to administer and maintain the technique, the increased number of patients with respiratory injuries, and the shortage of materials increase the risk of infection during these procedures (67). Furthermore, the use of a bladder catheter increases the risk of catheter-associated urinary tract infection (68), and a central venous catheter is associated with mortality in chronic hemodialysis patients with COVID-19 in Brazil (69). Additionally, SAPS3, a scoring system widely used for predicting in-hospital mortality, was able to discriminate between survivors and non-survivors in our study (17–19), and the highest concentration of deaths due to COVID-19 was in ICU patients with SAPS3 > 51.5.

In summary, this study reported the clinical profile of a low-income population admitted to the COVID-19 ICU at a reference hospital in the Southern Region of Bahia State, Brazil. Our data demonstrate that the use of a catheter (central venous, arterial, or bladder) was the main factor associated with death in COVID-19 patients. Although platelet count was not associated with the death of patients in the ICU, leukocyte count and biochemical parameters were valuable indicators of death. The SAPS3 presented the highest sensitivity (77.9%) and specificity (63.1%) for discriminating between survivors and non-survivors, with an AUC of 0.79. Lastly, we suggest that multi-laboratory parameters can be used to predict patient prognosis and guide healthcare teams toward more assertive clinical management, better resource allocation, and improved survival of patients admitted to COVID-19 in the ICU.

## 5. Conclusion

We identified some factors (epidemiological and laboratory) associated with a higher chance of death among patients with COVID-19 treated in the ICU. For example, patients aged 65 years or older, those with a prolonged ICU stay, and those who required catheter use were more likely to die of COVID-19. Identifying predictors of death is important for choosing the best clinical management and therapeutic approaches to patients to avoid or minimize unfavorable outcomes. Moreover, it is important that epidemiological and clinical laboratory data are available for decision-making purposes. Thus, by knowing the predictors of worse prognosis and having these data, clinicians can act early and with scientific evidence.

## Data availability statement

The original contributions presented in the study are included in the article/Supplementary material, further inquiries can be directed to the corresponding author/s.

## Ethics statement

The studies involving humans were approved by the Human Beings Research Ethics Committee of the State University of Santa Cruz, Ilhéus, Brazil. The studies were conducted in accordance with the local legislation and institutional requirements. The participants provided their written informed consent to participate in this study.

## Author contributions

LC, JG, CJ, and HP: conceptualization. MD’C, US, LC, and RF: methodology. RF, AC, CR, and AS: validation. MD’C and US: formal analysis. US, MD’C, and LC: data curation. MD’C, AC, CM, EG, SG, LM, and LC: original draft preparation. US, CJ, RR, RF, SG, AC, CR, and AS: writing review and editing. LC: supervision. All authors have read and agreed to the published version of the manuscript.
