# Supplementary material for: Relationship between clinical-epidemiological parameters and outcomes of patients with COVID-19 admitted to the intensive care unit: a report from a Brazilian hospital
Source: Front Public Health. 2023 Sep 22;11:1241444. doi: 10.3389/fpubh.2023.1241444 (PMC10556466; doi:10.3389/fpubh.2023.1241444)
Supplement: Supplementary file 1 [file Table_1.DOCX]

Supplementary Material

Clinical and Epidemiological Parameters of COVID-19 Patients and Their Correlation to Intensive Care Unit Patients' Prognoses in a Reference Hospital of the Southern Region of Bahia State, Brazil

Maisah Meyhr D’Carmo Sodré¹, Uener Ribeiro dos Santos¹, Heitor Portella Povoas², Júlio Lenin Guzmán², Caroline Junqueira^5,6,7^, Tayana Oliveira Trindade², Sandra Rocha Gadelha^1^, Carla Cristina Romano¹, Aline Oliveira da Conceição¹, Eduardo Gross^1^, Aline Silva^1^, Rachel Passos Rezende^1^, Renato Fontana^1^, Camila Pacheco Silveira Martins da Mata^3^, Lauro Juliano Marin^4^, Luciana Debortoli de Carvalho¹

***Correspondence:**

Luciana Debortoli de Carvalho

ldcarvalho@uesc.br


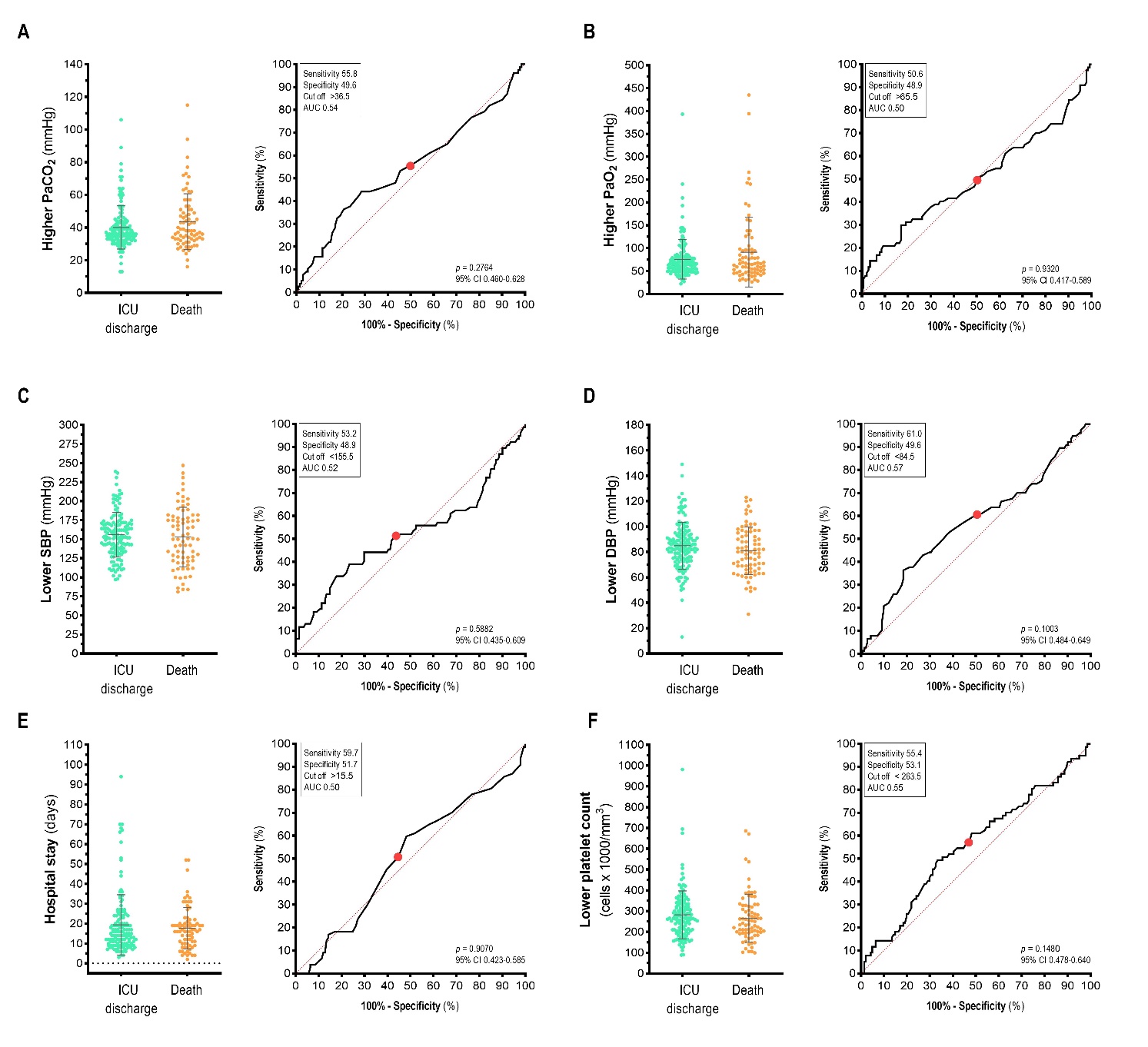


**Supplementary Figure S1.** Clinical parameters used to discriminate survivors and non-survivors with COVID-19 in the ICU from a reference hospital in the Southern Region of Bahia State, in Brazil. Analysis of variables (left) and area under the curve (AUC, right) for (**A**) Higher PaCO_2_, (**B**) PaO_2_, (**C**) lower systolic blood pressure, (**D**) lower diastolic blood pressure, (**E**) hospital stay, and (**F**) lower platelet count. The red point indicates the cut-off value. Mann-Whitney test. Data are presented as mean ± standard deviation. p *<* 0.05 were considered for statistical significance.
